# Supplementary material for: Evaluation of the Physico-Chemical Properties of Liposomes Assembled from Bioconjugates of Anisic Acid with Phosphatidylcholine
Source: Int J Mol Sci. 2021 Dec 5;22(23):13146. doi: 10.3390/ijms222313146 (PMC8658227; doi:10.3390/ijms222313146)
Supplement: Supplementary file 1 [file ijms-22-13146-s001.zip › ijms-1461876-supplementary.pdf]

**Table S1.** Assigned bands of ATR -FTIR spectra of DPPC film and for 1-ANISA-2-PA-PC/DPPC (1:5) and 1-PA-2-ANISA-PC/DPPC (1:5) systems at different temperatures.

| ATR spectra, wavenumbers ($cm^{-1}$) | | | |
|---|---|---|---|
| **Assignet*** | **DPPC** | **1-ANISA-2-PA-PC/DPPC** | **1-PA-2-ANISA-PC/DPPC** |
| | | **$25^0$C** | |
| $\delta(CH_2)$ | 1467.44 | 1467.35 | 1467.37 |
| $\gamma_r(CH_2)$ | 720.98 | 721.07 | 721.06 |
| $\nu_s$ (N-C) | 925.80 | 926.07 | 925.85 |
| $\nu_{as}$ $(N\text{-}C)_{ip}$ | 969.24 | 969.37 | 969.36 |
| $\nu_s$ (COP) | 1060.96 | 1062.57 | 1090.15 |
| $\nu_s(PO_2^-)$ | 1089.44 | 1090.21 | 1062.31 |
| $\nu_{as}(PO_2^-)$ | 1244.92 | 1244.10 | 1244.42 |
| $\nu$(C=O) | 1732.40 | 1731.22 | 1734.50 |
| | 1736.72 | 1735.35 | - |
| $\nu_s(CH_2)$ | 2849.36 | 2849.69 | 2849.44 |
| $\nu_s(CH_3)$ | 2873.17 | 2872.75 | 2873.11 |
| $\nu_{as}(CH_2)$ | 2916.46 | 2917.13 | 2916.56 |
| $\nu_{as}(CH_3)$ | 2955.72 | 2955.53 | 2955.71 |
| | | **$38^0$C** | |
| $\delta(CH_2)$ | 1467.50 | 1467.46 | 1467.69 |
| $\gamma_r(CH_2)$ | 720.95 | 721.12 | 720.97 |
| $\nu_s$ (N-C) | 924.70 | 925.35 | 925.31 |
| $\nu_{as}$ $(N\text{-}C)_{ip}$ | 968.80 | 968.91 | 969.02 |
| $\nu_s$ (COP) | 1062.34 | 1062.98 | 1062.74 |
| $\nu_s(PO_2^-)$ | 1089.29 | 1090.00 | 1089.88 |
| $\nu_{as}(PO_2^-)$ | 1245.23 | 1244.71 | 1245.51 |
| $\nu$(C=O) | 1732.59 | 1731.15 | - |
| | 1736.69 | 1735.46 | 1734.61 |
| $\nu_s(CH_2)$ | 2849.51 | 2849.54 | 2849.53 |
| $\nu_s(CH_3)$ | 2873.34 | 2873.24 | 2873.30 |
| $\nu_{as}(CH_2)$ | 2916.95 | 2916.95 | 2916.97 |
| $\nu_{as}(CH_3)$ | 2956.12 | 2956.42 | 2956.10 |
| | | **50°C** | |
| $\delta(CH_2)$ | 1467.39 | 1467.45 | 1464.88 |
| $\gamma_r(CH_2)$ | 720.02 | 720.71 | 720.68 |
| $\nu_s$ (N-C) | 925.95 | 924.28 | 924.32 |
| $\nu_{as}$ $(N\text{-}C)_{ip}$ | 967.77 | 968.38 | 968.41 |
| $\nu_s$ (COP) | 1058.40 | 1064.62 | 1064.25 |
| $\nu_s(PO_2^-)$ | 1087.28 | 1089.88 | 1089.94 |
| $\nu_{as}(PO_2^-)$ | 1244.96 | 1244.39 | 1245.41 |
| $\nu$(C=O) | 1722.82 | - | - |
| | 1736.48 | 1734.73 | 1734.61 |
| $\nu_s(CH_2)$ | 2849.34 | 2849.85 | 2849.71 |
| $\nu_s(CH_3)$ | 2872.84 | 2873.20 | 2873,38 |
| $\nu_{as}(CH_2)$ | 2916.43 | 2917.73 | 2917.47 |
| $\nu_{as}(CH_3)$ | 2955.32 | 2956.42 | 2956.41 |

* vibrations: δ – bending, γ - deformation; ν - stretching; r - rocking; s - symmetric; as – antisymmetric;

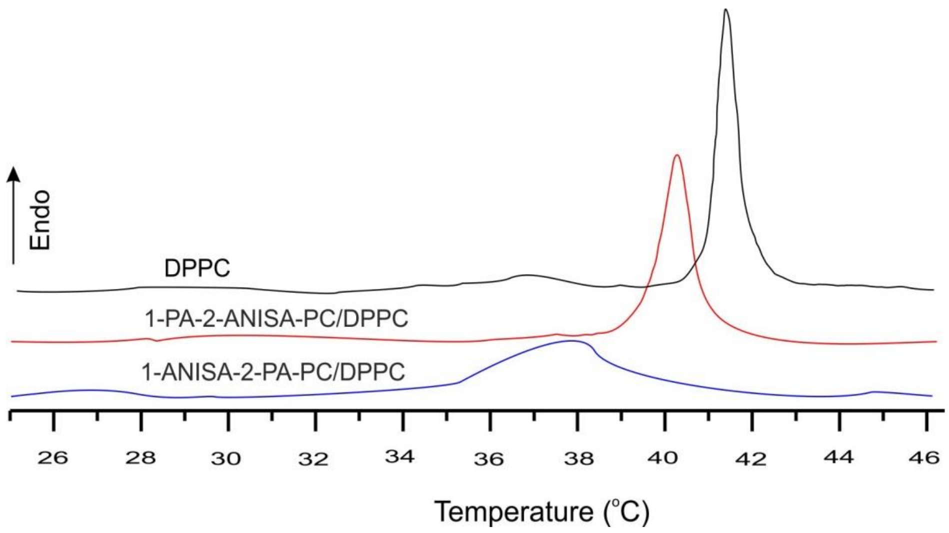


**Figure S1.** DSC thermogram of DPPC liposomes and new form of liposomes composed of DPPC and conjugates of anisic acid with phosphatidylcholine at molar ratio conjugates/DPPC 1:1
